# Infertility highlighted in International Brazilian Journal of Urology

**DOI:** 10.1590/S1677-5538.IBJU.2021.03.01

**Published:** 2021-03-25

**Affiliations:** 1 Universidade do Estado de Rio de Janeiro Unidade de Pesquisa Urogenital Rio de JaneiroRJ Brasil Unidade de Pesquisa Urogenital - Universidade do Estado de Rio de Janeiro - Uerj, Rio de Janeiro, RJ, Brasil; 2 Hospital Federal da Lagoa Serviço de Urologia Rio de JaneiroRJ Brasil Serviço de Urologia, Hospital Federal da Lagoa, Rio de Janeiro, RJ, Brasil

The May-June number of Int Braz J Urol, the 10th under my supervision, presents original contributions with a lot of interesting papers in different fields: Prostate Cancer, Male Infertility, Renal Cell Carcinoma, Urinary Stones, Testicular Cancer, Bladder Cancer, Nocturnal Enuresis, Penile Cancer, Male Health, LUTS, Neurogenic Bladder, Pediatric Urology, Sacral Neuromodulation and Covid-19 in Urology. The papers came from many different countries such as Brazil, USA, Turkey, China, Chile, Portugal, Israel, Canada, Italy and Iran, and as usual the editor´s comment highlights some of them.

In the present issue we present four important papers about Male Infertility. Dr. Xavier and colleagues from Brazil performed in page 495 (1) a nice review about the Semen quality from patients affected by seminomatous and non-seminomatous testicular tumor and concluded that more studies will be necessary to clarify the behavior of seminoma and non-seminoma tumors implicating the reproductive health of male patients. Dr. Lorenzini and colleagues from Brazil performed in page 544 (2) a nice study about the vasectomy re-reversal (VRR) effectiveness and whether specific parameters can be associated with its success and concluded that VRR was effective in restoring the obstruction in more than half of the patients. Furthermore, the presence of spermatozoa in the vas deferens fluid was the parameter associated with the VRR success and Dr. Pariz and colleagues from Brazil performed in page 617 (3) a nice report about dysplasia of the fibrous sheath with axonemal and centriolar defects combined with lack of mitochondrial activity as associated factors of ICSI failure in primary ciliary dyskinesia syndrome and concluded that modern andrology, its clinicians and field scientists have the power and the tools to limit these complications by adopting a more rigorous assessment, especially that of the male gamete. In particular, an assessment of the sperm functional aspects, the presence of ROS, LPO and integrity of the nucleus/DNA; not limited to severe infertility, whether due to asthenozoospermia, severe oligozoospermia, teratozoospermia, leukocytospermia or any chronic disease. The editor in chief would like to highlight the following works too:

Dr. Fantin and collegues from Brazil presented in page 484 (4) a nice systematic review about the role of salvage lymph node dissection in patients previously treated for prostate cancer and shows that the papers studied do not present high levels of evidence to draw strong conclusions. However, even if significant rates of biochemical recurrence are not evident in all studies, therapy directed to lymph node metastases may present good oncological results and postpone the onset of systemic therapy.

Dr. Monteiro and collegues from Brazil presented in page 515 (5) a nice study about a very important disease in Brazil: Penile Cancer and the authors studied the erectile function after partial penectomy for penile cancer and shows that partial penectomy due to penile cancer provides adequate local control of the disease, however, proper counselling is important especially in relation to ED consequences. Preservation of penile length yields to more optimal erectile recovery.

Dr. Rangel and collegues from Brazil presented in page 535 (6) a very interesting study about the quality of life in enuretic children and concluded that enuresis has a great impact in quality of life of children and that this impact is not related to the age or sex of the child.

Dr. Oliveira and collegues from Brazil presented in page 558 (7) a study about the a single center experience in the public health system in Prostate Cancer Screening in Brazil and concluded that the PSA-screening remains controversial in literature. In front of a huge miscegenated people and considering the big proportion of high-risk PCa, even in young men diagnosed with the disease, it is imperative to inform patients and health providers about these data particularities in Brazil.

Dr. Leite and collegues from Brazil presented in page 566 (8) a very interesting study about the influence of treatment access on survival of metastatic renal cell carcinoma in brazilian cancer center and concluded that patients with metastatic renal cell carcinoma treated via the the public health system in a Brazilian Cancer Center. had worse overall survival, possibly due to poorer prognosis at presentation and less drug access.

Dr. Lopes and collegues from Brazil presented in page 574 (9) a very important study about patients with encrusted ureteral stents can be treated by a single session combined endourological approach and concluded that the endoscopic combined approach in the supine position is a safe and feasible technique that allows removal of retained and encrusted stents in a single procedure. The Forgotten- Encrusted-Calcified (FECal) classification seems to be useful for surgical planning.

Dr. Bolat and collegues from Turkey presented in page 584 (10) a nice prospective randomized study about the Monopolar versus bipolar transurethral resection of lateral wall-located bladder cancer under obturator nerve block and concluded that during the treatment of lateral-wall located non-muscle invasive bladder cancers, either monopolar TUR (M-TURBT) or bipolar TUR (B-TURBT) can be safely and effectively performed by combining spinal anesthesia with obturator nerve block. Even so, it should be taken into consideration that low-grade postoperative hemorrhagic complications may occur in patients who undergo M-TURBT.

Dr. Falahatkar and collegues from Iran presented in page 596 (11) a very nice randomized controlled clinical trial about the effects of pregabalin, solifenacin and their combination therapy on ureteral double-J stent-related symptoms and concluded that the combination therapy of pregabalin and solifenacin has a significant effect on stent-related symptoms and is preferred over monotherapy of the respected medications.

Drs. Wang and Braga from Canada presented in page 610 (12) a very important surgical technique about the open distal ureteroureterostomy (UU) for ectopic ureters in infants with duplex systems and no vesicoureteral reflux under 6 months of age – This paper is the Cover in this number – and concluded that an open distal UU can be performed safely and effectively through a small incision in the inguinal crease in infants younger than 6 months of age, with minimal morbidity and short hospital stay. Its excellent cosmesis and minimally invasive nature are comparable to any robotic or laparoscopic procedures.

Dr. Ferenczi and collegues from USA presented in Expert Opinion secion in page 631 (13) a interesting report about COVID-19 screening and shows that pre-procedural COVID-19 testing is a scalable intervention that will provide a means to safely reimplement care for the Urologic community. Eventually, Urolo- gic surgical volume will need to expand nationwide in the setting of the ongoing COVID-19 pandemic and limited PPE. Universal COVID-19 screening of pre-operative patients represents a viable means to meet the needs of our patients.

The Editor-in-chief expects everyone to enjoy reading this very interesting issue of the International Brazilian Journal of Urology.
